# Side Effects of mRNA-Based COVID-19 Vaccines among Young Adults (18–30 Years Old): An Independent Post-Marketing Study

**DOI:** 10.3390/ph14101049

**Published:** 2021-10-15

**Authors:** Abanoub Riad, Andrea Pokorná, Jitka Klugarová, Natália Antalová, Lucia Kantorová, Michal Koščík, Miloslav Klugar

**Affiliations:** 1 Czech National Centre for Evidence-Based Healthcare and Knowledge Translation (Cochrane Czech Republic, Czech EBHC: JBI Centre of Excellence, Masaryk University GRADE Centre), Institute of Biostatistics and Analyses, Faculty of Medicine, Masaryk University, Kamenice 5, 625 00 Brno, Czech Republic; apokorna@med.muni.cz (A.P.); klugarova@med.muni.cz (J.K.); lucia.kantorova@mail.muni.cz (L.K.); klugar@med.muni.cz (M.K.); 2 Department of Public Health, Faculty of Medicine, Masaryk University, Kamenice 5, 625 00 Brno, Czech Republic; natalia.antalova@med.muni.cz (N.A.); koscik@med.muni.cz (M.K.); 3 Department of Nursing and Midwifery, Faculty of Medicine, Masaryk University, Kamenice 5, 625 00 Brno, Czech Republic

**Keywords:** BNT162 vaccine, COVID-19, Czech Republic, drug-related side effects and adverse reactions, mass vaccination, mRNA-1273 vaccine, phase IV, prevalence, young adult

## Abstract

Young adults had been widely perceived as a low-risk group for COVID-19 severity; therefore, they were deprioritised within the mass vaccination strategies as their prognosis of COVID-19 infection is relatively more favourable than older age groups. On the other hand, vaccination of this demographic group is indispensable to achieve herd immunity. A cross-sectional survey-based study was used to evaluate the side effects of mRNA-based COVID-19 vaccines among university students in the Czech Republic. The validated questionnaire was delivered in a digital form, and it consisted of demographic data; COVID-19 vaccine-related anamnesis; and local, systemic, orofacial, and skin-related side effects’ prevalence, onset, and duration. Out of the 539 included participants, 70.1% were females and 45.8% were <23 years old. The vast majority (95.2%) reported at least one side effect. The most common side effect was injection site pain (91.8%), followed by fatigue (62.5%), headache (36.4%), and muscle pain (34.9%). The majority of local side effects occurred after both doses (74.4%), while most systemic side effects occurred after the second dose only (56.2%). Most local (94.2%) and systemic (93.3%) side effects resolved within three days after vaccination. Females participants’ adjusted odds ratio (AOR) showed they were 2.566 (CI 95%: 1.103–5.970) times more likely to experience post-vaccination side effects, and the participants who received two doses reported an increased AOR of 1.896 (0.708–5.077) for experiencing side effects. The results of this study imply that mRNA-based COVID-19 vaccines are highly probably safe for young adults, and further studies are required to investigate the role of medical anamnesis, prior COVID-19 infection, and gender in side effects incidence.

## 1. Introduction

The outbreak of the novel coronavirus diseases (COVID-19) has imposed unprecedented challenges to health systems worldwide that has led to disrupted services provision, delayed diagnoses, and increased severity and morbidity of major killers, the non-communicable diseases (NCDs) [1,2,3,4,5,6]. Therefore, mass vaccination strategies are strongly mandated to achieve substantial levels of community immunity that can guarantee the vulnerable with NCDs are protected [7,8]. Apprehension of post-vaccination side effects has been depicted as a key barrier for vaccination by the World Health Organization (WHO) Strategic Advisory Group of Experts on Immunization (SAGE) [9,10]. This proposition has been repeatedly confirmed in the context of COVID-19 vaccines, especially among young adults. Khuc et al. (2021) found that concerns about potential side effects were significantly associated with COVID-19 vaccine hesitancy and rejection among Vietnamese youth [11]. Similarly, studies from the United States (US), Egypt, Portugal, China, and Japan concluded that aversion to side effects was associated with an increased risk of vaccine hesitancy among the youth population [12,13,14,15,16,17]. A recent global cross-sectional study of healthcare students (n = 6639) found that the low confidence in COVID-19 vaccines safety was a significant promoter of vaccine hesitancy [18].

In March 2020, Liao et al. published the first epidemiologic evidence of COVID-19’s impact on young adults (≤35 years old), where the vast majority of included cases exhibited mild forms of clinical severity and some of them were asymptomatic [19]. Since that time, asymptomatic young adults have been known to be able to transmit the severe acute respiratory syndrome coronavirus 2 (SARS-CoV-2) infection to their households [19]. Therefore, the nonpharmacologic measures in universities, other higher education institutions, and workplaces were deemed necessary to control the community transmission. Several studies emerged recently to demonstrate the negative impact of COVID-19 on the mental health of young adults, especially university students, who were dramatically shifted from campus education to remote learning with very minimal interpersonal communication and support [20,21,22,23,24].

The young adults have faced another challenge in trying return to the normal setting, which is why the WHO guideline recommends prioritising certain population groups to receive COVID-19 vaccines based on their empirically determined risk. Frontline healthcare workers, essential workers, older adults, and individuals with comorbidities were widely accepted as the priority groups in most countries, including the European Union (EU) member states [7]. However, though this policy has proven to be effective so far, it may have increased the levels of vaccine hesitancy among young adults inadvertently by giving them a false sense of protection as a low-risk group. Additionally, this policy led to an increased strain on the young adults’ return to normal settings due to long waits for their vaccinations. For example, the COVID-19 vaccine rollout began in the Czech Republic on 27 December 2020, and young adults (≤30 years old) had to wait over five months to start to register for vaccination on 4 June 2021 [25].

Though young adults have an empirically confirmed low risk of COVID-19 severity for known virus variants, they were found to be at increased risk of long-standing complications following the mild course of COVID-19 infection, which are referred to as (long COVID) [26]. Moreover, young adults represent a critical demographic group for achieving herd immunity through vaccination. Therefore, their attitudes towards receiving COVID-19 vaccines are of practical value for our battle against SARS-CoV-2. Heretofore, we identified a lack of evidence on the post-vaccination side effects of this particular group as they were conventionally combined with middle-aged adults in one cohort, which was consequently compared against the senior adults.

The overarching aim of this study was to evaluate the safety of the mRNA-based COVID-19 vaccines among the young adult population. Therefore, the primary objective was to estimate the prevalence, onset, and duration of the self-reported side effects following mRNA-based vaccine administration. The secondary objective was to evaluate the association between the post-vaccination reported side effects and their potential risk factors among the target population.

## 2. Materials and Methods

### 2.1. Design

This post-marketing (phase IV) trial was designed as a cross-sectional study targeting university students in the Czech Republic who received mRNA-based COVID-19 vaccines in the early months of 2021.

The study utilised a validated questionnaire created in a digital form and disseminated using KoBoToolbox version 2.021.03 (Harvard Humanitarian Initiative, Cambridge, MA, USA, 2021). The protocol of this study was registered a priori in the US National Library of Medicine (NLM) with the title (COVID-19 Vaccines Safety Tracking—CoVaST) and under the identifier NCT04834869 [27,28].

### 2.2. Participants

The target population was young adults aged between 18 and 30 years old; therefore, the full-time students enrolled in Czech universities were approached. Non-random sampling through the snowballing technique was used to recruit the study participants. The digital questionnaire was circulated through the students’ organisations, the students’ representatives of the universities’ academic senates, and the students’ representatives at the Council of Higher Education Institutions [29].

The recruitment took place from 21 April to 15 June 2021; at that time, people < 60 years old were not freely permitted to be vaccinated; however, the healthcare and social care workers, including the volunteering students, had been already halfway through their vaccination phase [30]. Therefore, the students who joined this study were primarily from these volunteering groups. 

**Figure 1:**
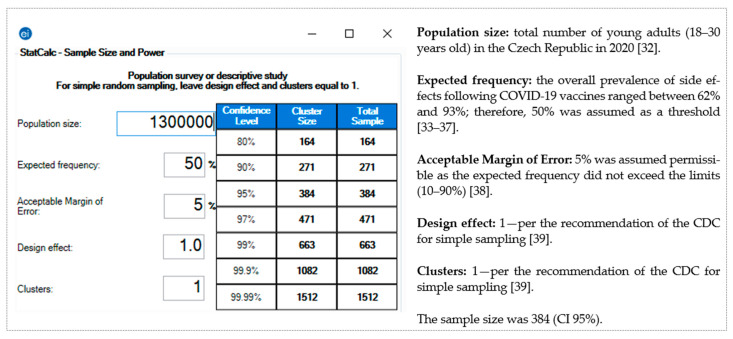
Sample size of the young adults (18–30 years old) in the Czech Republic—Epi-Info ^TM^ version 7.2.4.

The pragmatic sample size for this study was calculated using Epi-Info^TM^ version 7.2.4 (CDC, Atlanta, GA, USA, 2020) [31]. The following assumptions were used according to the total population size [32]: expected frequency, 50% [33,34,35,36,37]; margin of error, 5% [38]; confidence interval (CI), 95%; and the required sample was 384 [39] (Figure 1).

### 2.3. Instrument

The self-administered questionnaire consisted of sixteen closed-ended items, which were stratified into three categories: (a) demographic data, including gender, age, nationality, study year, field, and university; (b) COVID-19 vaccine-related anamnesis, including the type of vaccine, number of doses, and willingness to get the following doses; (c) post-vaccination side effects, including local, systemic, orofacial, and skin-related ones [25].

The questionnaire items were adapted from previous instruments that had been validated [33,34,35,36,37]. The results of the validation and reliability testing process have been published in detail elsewhere [33]. Two independent forward translators translated the instrument from English to Czech, then a panel of experts was appointed to evaluate the two Czech versions to resolve any discrepancies between them and create a common working version.

### 2.4. Ethics

The study was carried out in accordance with the Declaration of Helsinki, and it was reported according to the STROBE guidelines for cross-sectional studies [40,41]. The ethical approval was obtained from the ethics committee of the Faculty of Medicine, Masaryk University, with Ref No. 26/2021.

The participants had to provide their informed consent digitally before filling in the questionnaire, and they were able to withdraw at any time from the study without the need to justify their decision. The participants did not receive financial compensation or any other form of incentives to minimise both selection and information biases. The study data were stored and processed according to the European Union (EU) General Data Protection Directive (GDPR); therefore, no identifying personal data were collected from the participants [42].

### 2.5. Analysis

The Statistical Package for the Social Sciences (SPSS) version 27.0 (SPSS Inc., Chicago, IL, USA, 2020) was used to analyse the obtained dataset [43]. Before running any inferential tests, the normal distribution of the dependent variables was tested using Shapiro–Wilk test with a significance level (*Sig*.) of ≤0.05.

Primarily, descriptive statistics were carried out to present the demographic variables (gender, age, study year, field, and university), COVID-19 vaccine-related anamnesis (number of doses, duration between doses, and vaccine type), and post-vaccination side effects (prevalence, onset, and duration) using frequencies (*n*), percentages (%), mean (*μ*), and standard deviation (*SD*).

Consequently, inferential statistics were carried out to estimate the association between post-vaccination side effects and potential risk factors using the Chi-squared test (*χ*^2^), Fisher’s exact test if the expected frequency was less than 5, and the Mann–Whitney test (*U*). Binary logistic regression was used to evaluate the strength of association between the proposed predictors and the post-vaccination side effects. All the inferential tests were conducted with a significance level (*Sig*.) of ≤0.05.

## 3. Results

### 3.1. Demographic Characteristics

Five hundred and eighty-six students responded to the questionnaire, of which 15 were excluded because they were >30 years old. Further, thirty-two participants received viral vector-based vaccines; therefore, they were excluded from the downstream analysis while their data were pooled and analysed in [25].

Out of the remaining 539 included participants, 378 (70.1%) were females, and 360 (66.8%) were Czech nationals (66.8%). Their mean age was 22.86 ± 2.05 years; therefore, the age of 23 years was used as a cut-off in the downstream analyses. The most represented study field was medical and healthcare sciences (84%), followed by social sciences (5%) and arts and humanities (3.5%). The majority of participants were from Masaryk University (59.9%) and Charles University (30.6%). (Table 1).

### 3.2. COVID-19 Vaccine-Related Anamnesis

At the time of filling in the questionnaire, 86.3% of the participants had received two doses of the mRNA-based COVID-19 vaccines, and 13.7% received the first dose only. All the students (100%) who received the first dose were willing to receive the second dose. The mean duration between the first and second dose was 28.84 ± 15.17 days. While 92% received BTN162b2 COVID-19 vaccine, 8% received the mRNA-1273 COVID-19 vaccine. No significant differences between females and males were found in terms of the number of doses, duration between doses, and vaccine type. (Table 2).

### 3.3. Local Side Effects of mRNA-Based COVID-19 Vaccines

Overall, 92.4% of the participants reported at least one local side effect related to the injection site. Female participants (94.4%) had a significantly (*χ*^2^ = 7.957; *Sig.* = 0.005) higher level of local side effects prevalence compared to male participants (87.3%). The most common local side effect was injection site pain (91.8%), followed by injection site swelling (17.4%) and injection site redness (13.4%). Females (1.31 ± 0.77) had a significantly (*U* = 24,682; *Sig.* < 0.001) higher level of local side effects intensity compared to males (1.03 ± 0.59). The intensity was defined as the number of side effects reported by an individual, and it ranged between 0 and 3.

The ≥23-years-old participants (92.5%) had a similar level of local side effects compared to the <23-years-old participants (92.3%). Slovak students (95.5%) reported a higher level of local side effect prevalence than Czechs (90.8%). The healthcare students (94.3%) and the students who received two doses (93.5%) reported significantly (*χ*^2^ = 14.084 and 6.430; *Sig.* < 0.001 and =0.011) higher levels of local side effects compared to the non-healthcare students (82.6%) and the students who received one dose (85.1%).

While 74.4% of the participants who experienced local side effects reported that they occurred after both doses, 19.6% reported them after the first dose only, and 6% reported them after the second dose only. No significant differences were found in the onset of local side effects between females and males. The vast majority (94.2%) of local side effects resolved within three days after the vaccination—28.2% after the first day, 43.8% after the second day, and 22.2% after the third day. In general, the mean duration of local side effects was significantly different (*U* = 21,219.5; *Sig.* = 0.017) between females (2.15 ± 0.96) and males (1.93 ± 0.88). (Table 3).

The mean duration of local side effects was not significantly (*U* = 30,708 and 25,165.5; *Sig.* = 0.906 and 0.083) different among the ≥23-years-old participants (2.11 ± 1.01) vs. the <23-years-old participants (2.05 ± 0.86), and Czech students (2.15 ± 0.99) vs. Slovak students (1.96 ± 0.84). The mean duration of local side effects was significantly (*U* = 17,257.5 and 11,553; *Sig.* = 0.025 and 0.037) different among the healthcare students (2.05 ± 0.94) vs. the non-healthcare students (2.30 ± 0.97), and the students with two doses (2.06 ± 0.96) vs. the students with one dose (2.25 ± 0.82).

### 3.4. Systemic Side Effects of mRNA-Based COVID-19 Vaccines

**Figure 2:**
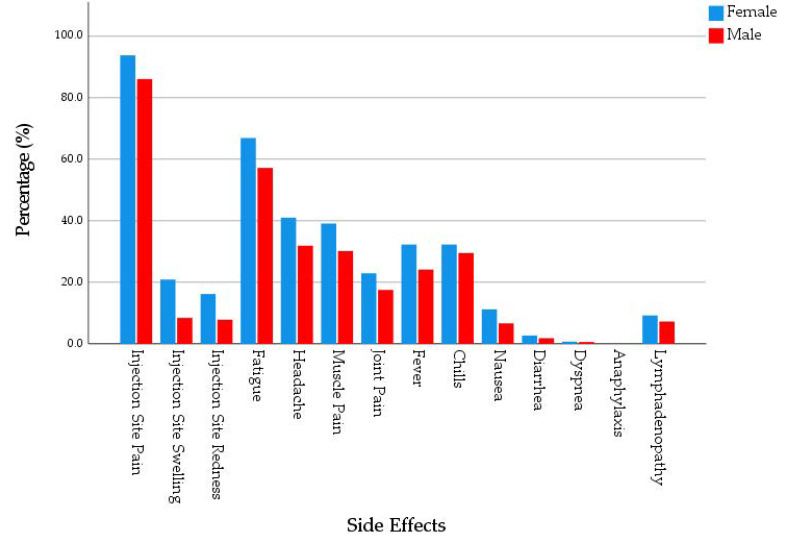
Side Effects of mRNA-based COVID-19 Vaccines Experienced by Young Adults (18–30 years old), Czech Republic, April–June 2021 (*n* = 539).

Overall, 72.5% of the participants reported at least one systemic side effect. Female participants (74.6%) had a slightly higher level of systemic side effects prevalence compared to male participants (67.7%). The most common systemic side effect was fatigue (62.5%), followed by headache (36.4%), muscle pain (34.9%), chills (29.9%), fever (27.3%), and joint pain (20.4%). Four participants, three of them were females, reported dyspnoea, and no participants reported anaphylaxis. Females had significantly higher prevalence of fatigue (65.6% vs. 55.7%), headache (39.2% vs. 29.7%), fever (29.6% vs. 21.5%), and nausea (11.1% vs. 5.1%) compared to males (*χ*^2^ = 4.680, 4.260, 3.698 and 4.818; *Sig.* = 0.031, 0.039, 0.054 and 0.028, respectively). Females (2.48 ± 2.19) had a significantly (*U* = 25,498.5; *Sig.* = 0.007) higher level of systemic side effects intensity compared to males (1.94 ± 2.08). The intensity was defined as the number of side effects reported by an individual, and it ranged between 0 and 11. (Figure 2).

The ≥23-years-old participants (73.6%), Czech students (71.7), and the healthcare students (72.4) had a similar level of systemic side effects prevalence compared to the <23-years-old participants (71.3%), Slovak students (74.3%), and the non-healthcare students (73.3). The students who received two doses (74.6%) reported a significantly (*χ*^2^ = 7.370; *Sig.* = 0.007) higher level of systemic side effects prevalence compared to the students who received one dose (59.5%).

While 56.2% of the participants who experienced systemic side effects reported that they occurred after the second dose only, 16.4% reported them after the first dose only and 27.4% reported them after both doses. The vast majority (93.3%) of systemic side effects resolved within three days after the vaccination—46.9% after the first day, 33.6% after the second day, and 12.8% after the third day. In general, the mean duration of systemic side effects was significantly longer (*U* = 13,160; *Sig.* = 0.050) among females (1.91 ± 1.13) compared to males (1.70 ± 1.03). (Table 4).

Only four participants, three females and one male, reported that their systemic side effects lasted for over a month, and their side effects included fatigue (75%), headache (25%), muscle pain (25%), and lymphadenopathy (50%). The onset of their systemic side effects was either after the second dose only (50%) or after both doses (50%).

The mean duration of systemic side effects was not significantly (*U* = 17,335, 15,894, and 6572.5; *Sig.* = 0.149, 0.245, and 0.169) different among the ≥23-years-old participants (1.78 ± 1.04) vs. the <23-years-old participants (1.94 ± 1.18), Czech students (1.89 ± 1.11) vs. Slovak students (1.78 ± 1.09), and the students who received two doses (1.84 ± 1.12) vs. those who received one dose (1.95 ± 0.925). The mean duration of local side effects was significantly (*U* = 12,378; *Sig.* = 0.003) different among the healthcare students (1.80 ± 1.10) vs. the non-healthcare students (2.15 ± 1.10).

### 3.5. Orofacial and Skin-Related Side Effects of mRNA-Based COVID-19 Vaccines

Overall, 3.5% of the participants reported at least one orofacial or skin-related side effect. Oral paraesthesia (1.3%) was the most common side effect, followed by oral ulcers (1.1%), taste disturbance (0.4%), skin rash (0.4%), and skin eruptions (0.4%). Only one female student (22 years old) reported Bell’s palsy following receiving BNT162b2. There was no significant difference between females and males in terms of orofacial and skin-related side effects prevalence or intensity. (Table 5).

### 3.6. Analgesic Drugs after mRNA-Based COVID-19 Vaccines

Out of the 539 participants, 165 (30.6%) reported using analgesic drugs after the vaccination to relieve their post-vaccination side effects. Females (34.7%) had a significantly (*χ*^2^ = 10.922; *Sig.* = 0.001) higher level of analgesics consumption compared to males (20.3%).

The most frequently used drug was acetaminophen (69.1%), through its common brand names *Paralen* (Opella Healthcare Czech s.r.o., Prague, Czech Republic) and *Panadol* (GlaxoSmithKline Consumer Healthcare Czech Republic s.r.o., Prague, Czech Republic). About one quarter of the participants consumed ibuprofen (25.5%) through its common brand names *Ibalgin* (Opella Healthcare Czech s.r.o., Prague, Czech Republic) and *Ibuprofen* (STADA PHARMA CZ s.r.o., Prague, Czech Republic) (Table 6).

Most side effects, both locally and systemically, were significantly associated with the use of analgesics, including injection site pain (33.1% vs. 2.3%; *χ*^2^ = 18.115; *Sig.* < 0.001), injection site swelling (39.4% vs. 28.8%; *χ*^2^ = 4.103; *Sig.* = 0.001), fatigue (43.3% vs. 9.4%; *χ*^2^ = 68.401; *Sig.* < 0.001), headache (55.6% vs. 16.3%; *χ*^2^ = 90.626; *Sig.* < 0.001), muscle pain (51.6% vs. 19.4%; *χ*^2^ = 59.844; *Sig.* < 0.001), joint pain (59.1% vs. 23.3%; *χ*^2^ = 52.770; *Sig.* < 0.001), fever (70.1% vs. 15.8%; *χ*^2^ = 148.137; *Sig.* < 0.001), chills (55.8% vs. 20.4%; *χ*^2^ = 65.411; *Sig.* < 0.001), nausea (56% vs. 28%; *χ*^2^ = 16.723; *Sig.* < 0.001), and lymphadenopathy (47.9% vs. 28.9%; *χ*^2^ = 7.428; *Sig.* = 0.006).

The use of analgesics was significantly (*U* = 35,768 and 51,414.5; *Sig.* < 0.001 and <0.001) associated with higher intensity levels of local side effects (1.39 ± 0.71 vs. 1.16 ± 0.72) and systemic side effects (4.05 ± 1.90 vs. 1.56 ± 1.82). Similarly, the use of analgesics was significantly (*U* = 31,547 and 21,385.5; *Sig.* = 0.003 and 0.005) associated with longer duration of local (2.25 ± 0.95 vs. 2.00 ± 0.93) and systemic side effects (1.95 ± 1.01 vs. 1.78 ± 1.16).

### 3.7. Risk Factors of Post-Vaccination Side Effects

Binary logistic regression revealed that females with an adjusted odds ratio (AOR) were 2.566 (CI 95%: 1.103–5.970; *Sig.* = 0.029) times more likely to experience post-vaccination side effects compared to males. The ≥23-years-old participants had an AOR of 1.791 (CI 95%: 0.775–4.139; *Sig.* = 0.173) for experiencing post-vaccination side effects compared to the <23-years-old participants. Similarly, Slovak students (AOR: 2.592; CI 95%: 0.842–7.979), healthcare students (AOR: 2.933; CI 95%: 1.100–7.825), the participants who received two doses (AOR: 1.896; CI 95%: 0.708–5.077), and the participants who received BNT16b2 (AOR: 1.389; CI 95%: 0.377–5.110) had higher adjusted ratios of experiencing post-vaccination side effects. (Table 7).

On analysing the potential risk factors of local side effects, the AOR for female participants showed that they were2.903 (CI 95%: 1.473–5.722; *Sig.* = 0.002) times more likely to experience local side effects compared to males. Similarly, the AOR of healthcare students showed that they were 3.542 (CI 95%: 1.545–7.712; *Sig.* = 0.003) times more likely to experience local side effects compared to non-healthcare students.

On analysing the potential risk factors of systemic side effects, the participants who received two doses had an AOR showing that they were 2.237 (CI 95%: 1.261–3.969; *Sig.* = 0.006) times more likely to experience systemic side effects than the participants who received one dose. (Table 8).

## 4. Discussion

In total, 95.2% of the participating young adults (18–30 years old) reported at least one side effect after vaccination against COVID-19 with mRNA-based vaccines. Although we were also collecting data about viral vector-based vaccines in the Czech Republic, we collected a very small sample size that would not contribute to the statistical analyses. The most common side effects of mRNA-based vaccines were injection site pain (91.8%), fatigue (62.5%), headache (36.4%), and muscle pain (34.9%). The majority of local side effects occurred after both doses (74.4%), while most systemic side effects occurred after the second dose only (56.2%). Most local (94.2%) and systemic (93.3%) side effects resolved within three days after vaccination.

All prior active surveillance studies of COVID-19 vaccines concluded that younger age groups had an increased risk of side effects incidence [33,34,35,36,37,44,45,46,47,48,49]. Mathioudakis et al. (2021) surveyed a sample of recently vaccinated individuals, mainly from the United Kingdom (UK) and Greece, using 60 years as a cut-off for their age-related analysis [45]. Their multivariate analyses confirmed a strong negative relationship between age and the self-reported side effects [45]. In a national cross-sectional study in the UK, Menni et al., 2021 found that the ≤55-years-old individuals had significantly higher levels of side effects prevalence, including injection site pain, headache, and fatigue, compared to the >55-years-old individuals [49]. This trend was found in both the mRNA-based (BNT162b2) and the viral vector-based vaccine (ChAdOx1 nCoV-19) [49].

Riad et al., (2021) examined a sample of healthcare workers from the Czech Republic who received the BNT162b2 vaccine and found that the ≤43-years-old group had significantly higher levels of general side effects [33]. Similar results were found in Jordan by Abu-Hammad et al., 2021 and in Malta by Cuschieri et al., (2021) among healthcare workers while using 45 years of age as a cut-off point [47,50].

In a randomised phase IV trial of mRNA-1273, young adults (18–30 years old) represented only 6.02% of the entire sample, thus indicating that this cohort was not an interesting population group for the investigators [44]. In the rest of the published post-marketing studies, there is a lack of age-stratified analyses; therefore, it is not possible to evaluate the safety profile of COVID-19 vaccines for young adults based on these studies [33,34,35,36,37,45,46,47,48,49].

On evaluating the phase III results of the BNT162b2 vaccine, published by the US Centre for Disease Control and Prevention (CDC), injection site pain (80.5%) among young and middle-aged adults (18–55 years old) was significantly (*χ*^2^ = 38.568; *Sig.* < 0.001) less prevalent than what was found in our participants (91.9%) who received the BNT162b2 vaccine [51]. Similarly, injection site swelling and injection site redness were significantly (*χ*^2^ = 77.591 and 49.899; *Sig.* < 0.001 and <0.001) more prevalent among our sample (16.7% and 13.1%, respectively) than in the manufacturer’s report (6% and 5.2%, respectively) [51].

Fatigue was significantly (*χ*^2^ = 13.775; *Sig.* < 0.001) more prevalent among our sample (61.9%) than in the manufacturer’s report (53.1%) [51]. Similarly, muscle pain (34.7% vs. 28.9%), fever (26% vs. 9.5%), chills (27.4% vs. 24.1%), and joint pain (20.2% vs. 16.2%) were more prevalent in our sample compared to the manufacturer’s report [51]. Contrarily, headache (46.6% vs. 36.5%) and diarrhoea (10.8% vs. 2.8%) were more prevalent in the manufacturer’s report than in our sample [51]. The analgesics consumption was slightly lower among our sample (29.8%) compared to the manufacturer’s report (30.1%) [51].

In our sample, females were at greater risk of experiencing post-vaccination side effects. In February 2021, the CDC published a report on the side effects of COVID-19 vaccines, where 72% of the reports were of females, while only 61% of the vaccine doses were administered to females [52]. This result is in agreement with the findings of Di Resta et al. (2021), where post-vaccination side effects were more frequent among female healthcare workers in Italy compared to their male colleagues [53]. They also found that females had significantly higher serological values, thus suggesting that the more frequent and more severe side effects experienced by females could be related to the more vigorous immune response they had developed [53].

While testosterone generally decreases the immune functions and increases, in particular, males’ susceptibility to viral infections, the physiological levels of oestrogen stimulate humoral responses to viral infections by activating antibody-producing cells [54,55]. The more potent immune response and the lower pain threshold of females are among the suggested propositions attempting to explain the gender-based differences in self-reported COVID-19 vaccine side effects [56,57]. Moreover, the sociocultural structure of femininity and masculinity may play another role in this issue, as females are more inclined to seek medical care than males, who may have several barriers to help-seeking behaviours [58].

In the past, the female gender was reportedly associated with a higher level of side effects prevalence after various viral vaccines, e.g., influenza, attenuated Japanese encephalitis, measles–mumps–rubella combination vaccine (MMR), and attenuated Dengue vaccines [56,59]. Halsey et al. (2013) found that females were four times more likely to report allergic reactions following H1N1 vaccination than males, and this difference was only prominent during the childbearing age and disappeared in the other age groups [60].

Di Resta et al. (2021) also found that antibody titre and side effects were decreasing with age, thus placing the young adults at a greater risk of more frequent and more severe side effects, especially the female youth [53]. On comparing adolescents (12–15 years old) and young adults (18–25 years old), the BNT162b2 vaccine was found to induce greater immune response among adolescents and almost the same safety profile and side effects prevalence [61].

Oral paraesthesia (1.3%) and oral ulcers (1.1%) were rarely reported by our participants, thus indicating that oral side effects among young adults might have a low prevalence. While the COVID-19 infection-related oral manifestations were reported by young adults as well as middle-aged and senior adults, all the reported cases of oral side effects following BNT162b2 and ChAdOx1 nCoV-19 vaccines belonged to middle-aged adults [62,63,64,65,66,67,68,69,70,71,72]. Skin rash (0.4%) and skin eruptions (0.4%) were also reported rarely by our participants and evidence on the predicted prevalence of the rare orofacial and skin-related side effects is still lacking [73].

### 4.1. Strengths

To the best of our knowledge, this is the first study to evaluate the self-reported side effects of young adults (18–30 years old) following COVID-19 vaccination in the post-marketing phase. The recruited sample of this study were university students (84% in healthcare) with a likely higher level of health literacy and scientific background that predisposes them to understand and fill out this kind of questionnaire reliably and properly.

The proportion of the participants who had received one dose was small and all of them expressed their interest to get the second dose regardless of the side effects they had experienced. Another strong point of this study is that it is one of the few studies that investigated the use of analgesics to manage the post-vaccination side effects.

### 4.2. Limitations

The first limitation of this study is the lack of information about the medical anamnesis of the participants, including chronic diseases and regular medications; nevertheless, this can be justified by the fact that the prevalence of chronic illnesses among this particular age group is supposedly very low and it would not have yielded a comparable sample size to explore the impact of pre-existing conditions and medications on the post-vaccination side effects.

The second limitation is the lack of information about any prior COVID-19 infection of the participants, though this can be justified by the fact that clinical presentation of COVID-19 in young adults tends to be mild or even asymptomatic, which may lead to underestimation of the impact of prior COVID-19 infection on the post-vaccination side effects [19]. Nevertheless, mild COVID-19 in young adults can lead to prolonged complications referred to as “long COVID”, which requires further investigation to establish the impact of COVID-19 vaccination on these complications [26].

The third limitation is the minuscule proportion of the participants who received the mRNA-1273 vaccine and viral vector-based COVID-19 vaccines in this sample; however, this can be justified by the fact that 81.95% of the administered shots in the Czech Republic were of BNT162b2 vaccine, 8.45% were of mRNA-1273, 8.44% were of ChAdOx1 nCoV-19, and 1.15% were of Ad26.COV2.S, as of 22 July 2021 [74]. Therefore, our sample was deemed to represent the actual situation in the Czech Republic, even if a non-random sampling technique was used.

The fourth limitation is the gender imbalance in our sample, as 70.1% of the participants were females. The latest report of the Czech Statistical Office (ČSÚ) revealed that 55.6% of public university students and 57.1% of private university students were females [32].

The fifth limitation is due to the snowballing technique (non-random sampling) that was used in recruiting the participants, as it may have led to self-selection bias, thus causing overestimation of the side effects prevalence. The students who experienced post-vaccination side effects may have been more inclined to respond to the questionnaire and pass it to their colleagues than those who did not experience post-vaccination side effects.

The sixth limitation is the lack of information about the severity of the solicited side effects in this survey, which had been omitted from this study because this type of question is subjected to a high risk of recall bias; therefore, future research on young adults’ side effects is recommended to investigate the severity of the mRNA-based COVID-19 vaccine side effects.

### 4.3. Implications

The findings of this study confirmed that immunisation of young adults against COVID-19 using mRNA-based vaccines is highly probably a safe process that needs to be accelerated to reach substantial levels of collective (herd) immunity. Future studies should evaluate the role of medical anamnesis and prior COVID-19 infection as they may have a role in the incidence and intensity of post-vaccination side effects among young adults, as the healthy young adults may have a stronger immune response, thus yielding more burdening side effects.

Future research needs to investigate the impact of COVID-19 vaccination on long COVID-19 complications among young adults. The gender-based differences of COVID-19 vaccine side effects require further investigation, where the female-related confounding variables like the menstrual cycle, pregnancy, and contraceptive consumption should be controlled. In addition, future research on COVID-19 vaccine safety should carry out age-stratified analyses, with a highlight on the young adult group (18–30 years old).

## 5. Conclusions

To the best of our knowledge, this is the first study to focus on the side effects of COVID-19 vaccines among young adults. In total, 95.2% of the participants reported at least one side effect after vaccination against COVID-19 with mRNA-based vaccines. The most common side effect was injection site pain (91.8%), followed by fatigue (62.5%), headache (36.4%), and muscle pain (34.9%).

The majority of local side effects occurred after both doses (74.4%), while most systemic side effects occurred after the second dose only (56.2%). Most local (94.2%) and systemic (93.3%) side effects resolved within three days after vaccination. The AOR of females participants showed that they were 2.566 (CI 95%: 1.103–5.970) times more likely to experience post-vaccination side effects, and the participants who received two doses had an increased AOR of 1.896 (0.708–5.077) for experiencing side effects.

The results of this study imply that mRNA-based COVID-19 vaccines are highly probably safe for young adults, and further studies are required to investigate the role of medical anamnesis, prior COVID-19 infection, and gender in side effects incidence.

## Figures and Tables

**Table 1:** Demographic characteristics of young adults (18–30 years old) receiving mRNA-based COVID-19 vaccines, Czech Republic, April–June 2021 (*n* = 539).

Variable	Outcome	Frequency (*n*)	Percentage (%)
**Gender**	Female	378	70.1%
	Male	158	29.3%
	Prefer not to say	3	0.6%
**Age**	<23 years old	247	45.8%
	≥23 years old	292	54.2%
**Nationality**	Czech	360	66.8%
	Slovak	179	33.2%
**Year**	1st Year	49	9.1%
	2nd Year	120	22.3%
	3rd Year	96	17.8%
	4th Year	111	20.6%
	5th Year	95	17.6%
	6th Year	47	8.7%
	Doctoral Candidate	21	3.9%
**Field**	Medical and Healthcare Sciences	453	84%
	Social Sciences	27	5%
	Arts and Humanities	19	3.5%
	Education and Social Care	16	3%
	Natural Sciences	8	1.5%
	Business and Economics	3	0.6%
	Technical Sciences	3	0.6%
	Agriculture, Forestry and Veterinary Sciences	2	0.4%
	Law	1	0.2%
	Military Sciences	1	0.2%
	Not Specified	6	1.1
	Healthcare Students (HCS)	453	84%
	Non-Healthcare Students (Non-HCS)	86	16%
**University**	Masaryk University	323	59.9%
	Charles University	165	30.6%
	Janáček Academy of Music in Brno	24	4.5%
	Palacký University Olomouc	18	3.3%
	Mendel University in Brno	2	0.4%
	Technical University of Ostrava	2	0.4%
	Czech University of Life Sciences in Prague	1	0.2%
	Prague University of Economics and Business	1	0.2%
	Other	3	0.6%

**Table 2:** mRNA-based COVID-19 vaccine-related anamnesis of young adults (18–30 years old), Czech Republic, April–June 2021 (*n* = 539).

Variable	Outcome	Female (*n* = 378)	Male (*n* = 158)	Total (*n* = 539)	*Sig.*
**Dose**	One Dose	49 (13%)	24 (15.2%)	74 (13.7%)	0.493
	Two Doses	329 (87%)	134 (84.8%)	465 (86.3%)	
**Duration**	Days	28.53 ± 14.01	29.53 ± 17.76	28.84 ± 15.17	0.831
**Type**	BNT162b2	345 (91.3%)	148 (93.7%)	496 (92%)	0.351
	mRNA-1273	33 (8.7%)	10 (6.3%)	43 (8%)	

Chi-squared test (*χ*^2^) and Mann–Whitney test (*U*) were used with a significance level (*Sig.*) of ≤0.05.

**Table 3:** Local side effects of young adults (18–30 years old) receiving mRNA-based COVID-19 vaccines, Czech Republic, April–June 2021 (*n* = 539).

Variable	Outcome	Female (*n* = 378)	Male (*n* = 158)	Total (*n* = 539)	*Sig.*
**Prevalence**	Injection Site Pain	355 (93.9%)	137 (86.7%)	495 (91.8%)	**0.006**
	Injection Site Swelling	81 (21.4%)	13 (8.2%)	94 (17.4%)	**<0.001**
	Injection Site Redness	60 (15.9%)	12 (7.6%)	72 (13.4%)	**0.010**
	Total (*n*)	357 (94.4%)	138 (87.3%)	498 (92.4%)	**0.005**
**Intensity**	(0–3)	1.31 ± 0.77	1.03 ± 0.59	1.23 ± 0.73	**<0.001**
**Onset**	After 1st Dose	58 (18.6%)	27 (22.7%)	85 (19.6%)	0.339
	After 2nd Dose	19 (6.1%)	7 (5.9%)	26 (6%)	0.936
	After Both Doses	235 (75.3%)	85 (71.4%)	322 (74.4%)	0.409
**Duration**	One Day: 1	91 (25.6%)	47 (34.3%)	140 (28.2%)	**0.053**
	Two Days: 2	157 (44.1%)	60 (43.8%)	217 (43.8%)	0.951
	Three Days: 3	83 (23.3%)	26 (19%)	110 (22.2%)	0.299
	Five Days: 4	17 (4.8%)	2 (1.5%)	19 (3.8%)	0.087
	One Week: 5	5 (1.4%)	1 (0.7%)	6 (1.2%)	1.000 *
	>One Week: 6	3 (0.8%)	1 (0.7%)	4 (0.8%)	1.000 *
	Total (1–6)	2.15 ± 0.96	1.93 ± 0.88	2.08 ± 0.94	**0.017**

Chi-squared test (*χ*^2^), Fisher’s exact test (*), and Mann–Whitney test (*U*) were used with a significance level (*Sig.*) of ≤0.05.

**Table 4:** Systemic side effects of young adults (18–30 years old) receiving mRNA-based COVID-19 vaccines, Czech Republic, April–June 2021 (*n* = 539).

Variable	Outcome	Female (*n* = 378)	Male (*n* = 158)	Total (*n* = 539)	*Sig.*
**Prevalence**	Fatigue	248 (65.6%)	88 (55.7%)	337 (62.5%)	**0.031**
	Headache	148 (39.2%)	47 (29.7%)	196 (36.4%)	**0.039**
	Muscle Pain	140 (37%)	46 (29.1%)	188 (34.9%)	0.079
	Joint Pain	83 (22%)	26 (16.5%)	110 (20.4%)	0.149
	Fever	112 (29.6%)	34 (21.5%)	147 (27.3%)	**0.054**
	Chills	113 (29.9%)	42 (26.6%)	156 (28.9%)	0.441
	Nausea	42 (11.1%)	8 (5.1%)	50 (9.3%)	**0.028**
	Diarrhoea	11 (2.9%)	3 (1.9%)	15 (2.8%)	0.767 *
	Dyspnoea	3 (0.8%)	1 (0.6%)	4 (0.7%)	1.000 *
	Anaphylaxis	0 (0%)	0 (0%)	0 (0%)	*N/A*
	Lymphadenopathy	36 (9.5%)	12 (7.6%)	48 (8.9%)	0.476
	Total (*n*)	282 (74.6%)	107 (67.7%)	391 (72.5%)	0.103
**Intensity**	(0–11)	2.48 ± 2.19	1.94 ± 2.08	2.32 ± 2.17	**0.007**
**Onset**	After 1st Dose	35 (14%)	22 (22.9%)	57 (16.4%)	**0.045**
	After 2nd Dose	144 (57.6%)	51 (53.1%)	195 (56.2%)	0.452
	After Both Doses	71 (28.4%)	23 (24%)	95 (27.4%)	0.406
**Duration**	One Day: 1	123 (43.6%)	59 (55.7%)	183 (46.9%)	0.034
	Two Days: 2	102 (36.2%)	29 (27.4%)	131 (33.6%)	0.102
	Three Days: 3	35 (12.4%)	14 (13.2%)	50 (12.8%)	0.833
	Five Days: 4	12 (4.3%)	2 (1.9%)	14 (3.6%)	0.367 *
	One Week: 5	5 (1.8%)	0 (0%)	5 (1.3%)	0.329 *
	>One Week: 6	2 (0.7%)	1 (0.9%)	3 (0.8%)	1.000 *
	>One Month: 7	3 (1.1%)	1 (0.9%)	4 (1%)	1.000 *
	Total (1–7)	1.91 ± 1.13	1.70 ± 1.03	1.85 ± 1.10	**0.050**

Chi-squared test (*χ*^2^), Fisher’s exact test (*), and Mann–Whitney test (*U*) were used with a significance level (*Sig.*) of ≤0.05.

**Table 5:** Orofacial and skin-related side effects of young adults (18–30 years old) receiving mRNA-based COVID-19 vaccines, Czech Republic, April–June 2021 (*n* = 539).

Variable	Outcome	Female (*n* = 378)	Male (*n* = 158)	Total (*n* = 539)	*Sig.*
**Prevalence**	Taste Disturbance	2 (0.5%)	0 (0%)	2 (0.4%)	1.000 *
	Oral Paraesthesia	5 (1.3%)	1 (0.6%)	7 (1.3%)	0.676 *
	Oral Ulcers	5 (1.3%)	1 (0.6%)	6 (1.1%)	0.676 *
	Bell’s Palsy	1 (0.3%)	0 (0%)	1 (0.2%)	1.000 *
	Skin Rash	1 (0.3%)	1 (0.6%)	2 (0.4%)	0.503 *
	Skin Eruption	2 (0.5%)	0 (0%)	2 (0.4%)	1.000 *
	Total (*n*)	15 (4%)	3 (1.9%)	19 (3.5%)	0.225
**Intensity**	(0–6)	0.4 ± 0.21	0.2 ± 0.14	0.4 ± 0.20	0.225

Chi-squared test (*χ*^2^), Fisher’s exact test (*), and Mann–Whitney test (*U*) were used with a significance level (*Sig.*) of ≤0.05.

**Table 6:** Analgesics used by young adults (18–30 years old) receiving mRNA-based COVID-19 vaccines, Czech Republic, April–June 2021 (*n* = 539).

Variable	Outcome	Female (*n* = 378)	Male (*n* = 158)	Total (*n* = 539)	*Sig.*
**Drug**	Ibuprofen	34 (26%)	7 (21.9%)	42 (25.5%)	0.634
	Acetaminophen	90 (68.7%)	23 (71.9%)	114 (69.1%)	0.727
	Other	14 (10.7%)	3 (9.4%)	17 (10.3%)	1.000 *
	Total (*n*)	131 (34.7%)	32 (20.3%)	165 (30.6%)	**0.001**

Chi-squared test (*χ*^2^) and Fisher’s exact test (*) were used with a significance level (*Sig.*) of ≤ 0.05. Significant values are in bold font.

**Table 7:** Predictors of mRNA-based COVID-19 vaccines side effects (general side effects) experienced by young adults (18–30 years old), Czech Republic, April–June 2021 (*n* = 539).

Predictor	B (SE)	AOR (CI 95%)	*Sig*.
Female (vs. Male)	0.942 (0.431)	2.566 (1.103–5.970)	**0.029**
≥23 years old (vs. <23 years old)	0.583 (0.427)	1.791 (0.775–4.139)	0.173
Slovak (vs. Czech)	0.952 (0.574)	2.592 (0.842–7.979)	0.097
HCS (vs. Non-HCS)	1.076 (0.501)	2.933 (1.100–7.825)	**0.032**
2 Doses (vs. 1 Dose)	0.640 (0.502)	1.896 (0.708–5.077)	0.203
BNT162b2 (vs. mRNA-1273)	0.328 (0.665)	1.389 (0.377–5.110)	0.621

Adjusted logistic regression was used with a significance level (*Sig.*) of ≤0.05. Significant values are in bold font.

**Table 8:** Predictors of mRNA-based COVID-19 vaccines side effects (local and systemic side effects) experienced by young adults (18–30 years old), Czech Republic, April–June 2021 (*n* = 539).

Predictor	Local Side Effects			Systemic Side Effects		
	B (SE)	AOR (CI 95%)	*Sig*.	B (SE)	AOR (CI 95%)	*Sig*.
Female (vs. Male)	1.066 (0.346)	2.903 (1.473–5.722)	**0.002**	0.303 (0.210)	1.353 (0.896–2.043)	0.150
≥23 years old (vs. <23 years old)	0.257 (0.346)	1.293 (0.657–2.547)	0.457	0.098 (0.199)	1.103 (0.747–1.629)	0.621
Slovak (vs. Czech)	0.660 (0.425)	1.934 (0.841–4.448)	0.121	0.086 (0.214)	1.090 (0.716–1.659)	0.687
HCS (vs. Non-HCS)	1.239 (0.410)	3.452 (1.545–7.712)	**0.003**	-0.328 (0.304)	0.720 (0.397–1.307)	0.280
2 Doses (vs. 1 Dose)	0.253 (0.439)	1.288 (0.545–3.042)	0.564	0.805 (0.292)	2.237 (1.261–3.969)	**0.006**
BNT162b2 (vs. mRNA-1273)	0.122 (0.575)	1.130 (0.366–3.485)	0.832	-0.219 (0.383)	0.804 (0.380–1.701)	0.568

Adjusted logistic regression was used with a significance level (*Sig.*) of ≤0.05. Significant values are in bold font.

## Data Availability

The data that support the findings of this study are available from the corresponding author upon reasonable request.
